# A lateral flow immunochromatographic strip test for rapid detection of hexoestrol in fish samples

**DOI:** 10.1098/rsos.180504

**Published:** 2018-08-29

**Authors:** Xingdong Yang, Zhongke Sun, Fengshou Tian, Guochao Jia, Jifei Yang, Xiaofei Hu

**Affiliations:** 1 Institute of Food and Drug Inspection, Zhoukou Normal University, Zhoukou 466001, People's Republic of China; 2 Key Laboratory of Animal Immunology of the Ministry of Agriculture, Henan Provincial Key Laboratory of Animal Immunology, Henan Academy of Agricultural Sciences, Zhengzhou 450002, People's Republic of China; 3 School of Medical Instrument and Food Engineering, University of Shanghai for Science and Technology, Shanghai 200093, People's Republic of China

**Keywords:** hexoestrol, immunoassay, colloidal gold, lateral flow immunochromatographic strip test (LFIST), rapid test

## Abstract

A lateral flow immunochromatographic strip test was developed for rapid and sensitive on-site detection of hexoestrol (HES) residues in fish samples with colloidal gold labelling of the anti-HES monoclonal antibody. The strip is composed of a sample pad, a conjugate reagent pad, an absorbent pad and a test membrane containing a control line and a test line. The sensitivity (half inhibitory concentration, IC_50_) of the strip in the detection of fish extract samples was confirmed to be 1.86 µg kg^−1^, and the limit of detection value was 0.62 µg kg^−1^. For intra-assay and inter-assay reproducibility, recoveries of HES-spiked samples ranged from 86.3% to 92.3% and 85.8% to 93.4%, coefficients of variation were 2.91–4.64% and 4.24–5.17%, respectively. High-performance liquid chromatography was employed to confirm the performance of the strip. The strip test takes less than 10 min, and thus provides a repaid method for on-site detection of HES residues.

## 1. Introduction

Hexoestrol (HES, 4,4'-(1,2-diethyl-1,2-ethanediyl) bisphenol), also known as hexoestrolum, is a kind of synthetic non-steroidal oestrogen, which can maintain the female character of the organism, promote the growth and development of reproductive organs, and adjust a positive and negative feedback on the hypothalamus and pituitary gland [1,2]. It has been mainly used in clinics for treatment of amenorrhoea, uterine hypoplasia, dysfunctional uterine bleeding, menopausal syndrome, senile vaginitis and prostatic cancer [3–6]. Though the use of HES in animals is banned in many countries including China, the USA and many European countries, unfortunately, HES has been illegally used in animal production as a growth-promoting additive for increasing feed conversion rate on account of the assimilation of protein and for its cost-efficiency [7]. Subsequently, the accumulation of β-agonist residues with long half-life in the meat of HES-treated animals could result in genital anomalies in human beings, and make women contract cancer through the food chain [8,9]. Histological lesions of liver and kidney, precocious puberty and boy feminization characterize the clinical profile of exposure to HES in humans [10]. Therefore, developing new methods for monitoring HES is necessary and important.

Traditional methods for the determination of HES include high-performance liquid chromatography (HPLC), gas chromatography–mass spectrometry (GC–MS) and high performance liquid chromatography–mass spectrometry (HPLC-MS) [11–14]. However, these methods require extensive sample preparation, expensive instruments and professional operation. Alternatively, enzyme-linked immunosorbent assay (ELISA) has been successfully developed for screening HES in urine or tissues [15,16], but, ELISA also needs incubation and washing steps and is mainly confined to laboratories. Since the development of the lateral flow immunochromatographic strip test (LFIST) for on-site detection of the concentrations of biotech crops in samples of ground grain [17], it has become a more popular method for the qualitative or semi-quantitative detection of various analytes [18–22] for its advantages of a one-step process (only addition of specimen), rapidity (less than 5 min) and cost-effectiveness [23,24].

In this paper, we describe the development of an LFIST for the rapid detection of HES residues in fish using an anti-HES monoclonal antibody (mAb). The LFIST employs a competitive format in which HES in the sample competes with HES-bovine serum albumin (BSa) on the test line for binding to the limited amount of colloidal gold-labelled anti-HES mAb, and is suitable for on-spot screening of large fish samples.

## 2. Material and methods

### 2.1. Reagents, material and apparatus

HES, RPMI-1640, hypoxanthine aminopterin thymidine (HAT), a mouse mAb isotyping kit and hypoxanthine thymidine (HT) were bought from Sigma (St Louis, MO, USA); PEG1500 (Mannheim, Germany); 1-(3-(dimethylamino) propyl)-3-ethylcarbodiimide hydrochloricde (EDC), *N*-hydroxy succinimide (NHS) (Fluka, China); BSA, ovalbumin (OVA), Freund's complete adjuvant (FCA) and Freund's incomplete adjuvant (FIA) were obtained from BDH (VWR International Ltd). ELISA plates and culture plates were bought from Nunc (Roskilde, Denmark); nitrocellulose (NC) membrane, glass fibre and absorbent pad were purchased from Millipore (Bedford, MA, USA); 4-bromobutyric acid ethyl ester, Goat anti-mouse IgG antibody (GaMIgG-HRP), *N, N*-dimethylformamide (DMF) (Shanghai, China); seven-week-old female BALB/c mice were acquired from the SPF standard Laboratory Animal Center (Zhengzhou University, China); GS-NS0 myeloma cells were donated by the institute for Animal Health of Great Britain; all other reagents and solvents were of analytical grade or higher in this study.

The Multifuge X1R high-speed refrigerated centrifuge was obtained from Thermo Scientific (Osterode, Germany); the XYZ Biostrip Dispenser, CM4000 Cutter, and TSR3000 membrane strip reader were purchased from Bio-Dot (Richmond, CA, USA); the Model 450 Microplate Reade (Richmond, CA, USA). The HPLC (L600) was obtained from Purkinje General Instrument Co., Ltd (Beijing, China).

### 2.2. Preparation of artificial antigens

Structure modification of HES was performed in accordance with the instruction described elsewhere [25] with minor modification, namely, under the protection of nitrogen stream, 550 mg of HES and 1.9 g of K_2_CO_3_–Al_2_O_3_ carrier reagent were dissolved in 20 ml of anhydrous acetone and 0.2 ml 4-bromobutyric acid ethyl ester, and the solution was heated in the dark for 12 h. The solid was removed by filtering. The filtrate was concentrated by rotary evaporation to about 5 ml in volume. The product was separated by thin-layer chromatography (TLC) using a chloroform–methanol mixture (95 : 5, v/v). The silica gel plate of the product was extracted with methanol and the solvent of the film was evaporated to achieve hexoestrol-mono-ether-butyrate-ethyl (HES-MEBE). The HES-MEBE was saponified and hydrolysed; the product was HES-mono-caroxyl-propyl-ethyl (HÈS-MCPE).

An active ester method was used to prepare immunogen (HES-BSA) and coating antigen (HES-OVA) [26]. Briefly, 35.0 mg of HES-MCPE, 12 mg of NHS and 20 mg of EDC were dissolved in 1 ml of DMF for 4 h at room temperature (RT). Sixty-six milligrams of BSA dissolved in 2.0 ml of phosphate buffered saline (PBS) were added to the above solution and stirred for 12 h at 4°C. The reaction product was purified by dialysis against 1 l of PBS for nine changes to remove the uncoupled free hapten and stored at −20°C. The conjugation ratio was characterized by UV.

### 2.3. Production of monoclonal antibody against hexoestrol

#### 2.3.1. Immunization of mice

Six seven-week-old BALB/c female mice were injected subcutaneously in back with immunogen (HES-BSA). The first immunization dose was 80 µg HES-BSA emulsified with an equal dosage of FCA. Five weeks later, four immunizations were given at three week intervals with the same amount of HES-BSA emulsified with the same dosage of FIA. Antisera were collected at two weeks after the five immunizations and were screened for anti-HES activity by competitive inhibition ELISA (*ci-*ELISA). One mouse that produced the highest anti-HES activity was used for fusion. The mouse received a final intraperitoneal injection of 80 µg of the conjugation in PBS. After 3 days, the spleen of the super-immune mouse was excised for cell fusion.

#### 2.3.2. Cell fusion and hybridoma screening

Hybridomas secreting anti-HES antibodies were generated according to a previous study [27]. First, PEG1500 was used to carry out fusion of NS0 cells and the spleen cell from the intraperitoneal injection of the mouse. The fused cells, mouse peritoneal macrophages and the selective HAT medium were then allotted into ELISA plates, respectively, and were incubated together. Nine days later, supernatants of hybridoma colonies were gleaned and detected by indirect ELISA antibody binding to HES. The positive wells of hybridoma cells were subcloned by limiting dilutions. Anti-HES mAb were produced using the mouse ascites method. The Ig subtypes of the mAb were determined by a commercialized mouse antibody isotype kit [28]. The affinity (*K*_a_) of mAb was determined by indirect ELISA [29].

#### 2.3.3. Indirect enzyme-linked immunosorbent assay and competitive inhibition enzyme-linked immunosorbent assay

Indirect ELISA was performed as follows: 96-well ELISA plates were coated with HES-OVA (detecting antigen) in 0.05 mol l^−1^ carbonate–bicarbonate buffer (pH 9.6) and incubated for 2 h at 37°C. Then the wells were blocked with 5% skimmed milk at 37°C for 1 h. Twofold serially diluted serum samples or cell culture supernatant was added and incubated at 37°C for 30 min. GaMIgG-HRP was added and incubated for another 30 min at 37°C. The optical densities (ODs) were measured at 450 nm with a microplate reader after colour development with TMB (3,3′, 5,5;-tetramethylbenzidine) chromogen solution and termination with 2 mol l^−1^ H_2_SO_4_. The *ci-*ELISA was carried out as follows: ELISA plates were coated as described above, and serially diluted free HES in PBS was then added together with the antibody and incubated for 30 min at 37°C. The following procedures were identical to those employed for indirect ELISA. During the steps, TBST (TBS containing 0.05% Tween 20 (v/v), pH 7.4) was used as a wash buffer to remove unbound antigens or antibodies.

### 2.4. Production of colloidal gold-labelled anti-hexoestrol monoclonal antibody

Colloidal gold nanoparticles (24 nm diameters) were made by reduction in gold chloride with 1% sodium citrate. The 100 µl colloidal gold was adjusted to pH 8.2 by 0.2 mol l^−1^ K_2_CO_3_, mixed with 40 µl of different concentrations of anti-HES mAb solution in a microplate under RT for 15 min. One hundred microlitres of 1 mol l^−1^ sodium chloride solution were added to the mixture. The colour of the micropore changed from blue to red as the concentration of mAb increased. The lowest concentration of anti-HES mAb solution did not change colour.

One hundred and twenty microlitres of optimal concentration of mAb solution were mixed with 12 ml of colloidal gold solution at RT, 20 min later, then 1 ml of 10% BSA solution in 0.02 mol l^−1^ Na_2_B_4_O_7_·10H_2_O solution was added to the mixture and stirred at RT for another 12 min. The labelled mAb was centrifuged at 15 000*g* at 4°C for 30 min. The supernatant was discarded. The precipitate was washed two times with 0.02 mol l^−1^ sodium borate (pH 9.0) containing 0.1% NaN_3_ and1% BSA. The colloidal gold-labelled mAb were stored in the 0.02 mol l^−1^ sodium borate buffer at 4°C.

### 2.5. Assembly of the strip

#### 2.5.1. Immobilization of capture reagents

HES-BSA and goat anti-mouse IgG were sprayed in the NC membrane in the form of dots at 1 µl cm^−1^ as the test and control lines (T and C lines), respectively. The T and C lines were both set at 5 mm separation from the centre of the NC membrane, and were dried for 20 min at 37°C. The final stage of the NC membrane was stored in a dry, closed environment.

#### 2.5.2. Manufacture of conjugate pad, sample pad and absorbent pad

One millilitre of colloidal gold-labelled mAb to HES was added in 2 ml of sodium borate buffer to prepare the conjugate solution. A conjugate pad was produced by dipping 7 × 300 mm glass fibre (Millipore) in the conjugate solution. After drying for 50 min at 37°C, the pad was stored at 4°C.

Sample and absorbent pads were composed of non-woven material (Millipore). Sample pad (15 × 300 mm) were soaked in the PBS (pH 7.2) buffer containing 0.1% (w/v) NaN_3_, 0.02 mol l^−1^ sodium borate, 2.0% (w/v) sucrose, and 2.0% (w/v) BSA, dried for 30 min at 50°C, stored at RT and kept in a dry, airtight environment.

The mentioned materials, absorption pad (40 × 300 mm) and support plate (75 × 300 mm) were installed according to the procedure described by Yang *et al*. [29].

#### 2.5.3. Basic test principle and process

The LFIST for detecting HES in fish samples is based on a competitive format. The anti-HES mAb labelled with colloidal gold was used as a probe. HES-BSA conjugates and goat anti-mouse IgG were dispensed onto the nitrocellulose membrane as the T line and C line, respectively. During detection applied to the strip, free HES in the sample would compete with HES-BSA on the test line for binding to mAb-gold conjugate. The colour of the test line is in a reverse relationship with the concentration of HES in the sample. For qualitative and semi-quantitative detection, the colour of the test line can be evaluated directly by the naked eye. For quantitative assay, the OD of a test line can be measured with a test strip reader, and according to the regression equation from the standard curve, the level of the HES residue can be calculated [30–33].

#### 2.5.4. Sample pretreatment for lateral flow immunochromatographic strip test

The fish samples from the market were identified to be negative by HPLC-MS. Six grams of negative fish meat (carassius, contains no HES) were homogenized, mixed with 12 ml of methanol, vortexed vigorously for 1 min, shocked for 10 min and centrifuged at 4000*g* for 5 min. The supernatant (1200 µl) was evaporated at 60°C under a gentle flow of nitrogen. The residue obtained was resuspended in 600 µl of the mixed solution PBS and methanol (4 : 1, V/V) as negative samples [34,35].

### 2.6. Evaluation of performance of lateral flow immunochromatographic strip test

The negative fish samples were spiked with 0, 1.0, 2.0, 4.0, 8.0, 16.0 and 32.0 ng ml^−1^ of HES. The sensitivity of the LFIST was determined by detection of the mentioned series of seven different HES concentrations and each concentration was performed in triplicate assays. The relative optical density (ROD) of the T line was obtained with a TSR3000 membrane strip reader (Bio-Dot) for constructing the standard curve. Then, the half inhibitory concentration (IC_50_) could be deduced from the regression equation. In order to determine the limit of detection (LOD) of the strip by the naked eye, two additional samples with 40.0 and 48.0 ng ml^−1^ of HES were also employed besides the aforementioned samples.

The specificity of the test strip was identified by cross-reactivity (CR), adding other similar competitors to the negative fish samples, including diethylstilbestrol, dienestrol, bisphenol A, estradiol, estriol and provera at a concentration of 1 µg ml^−1^. The CR was calculated by the equation: CR (%) = ((IC_50_ of HES)/IC_50_ of the other competitors) × 100%.

To estimate the accuracy, fish samples containing 4, 12 and 24 µg kg^−1^ of HES were tested by one batch of the test strips for replication analysis (*n* = 6). For inter-assay precision, three batches of the test strips were used to detect the given samples in triplicate. Accuracy and precision were expressed as coefficients of variation (CV).

### 2.7. Comparison of lateral flow immunochromatographic strip test and high-performance liquid chromatography

Fish samples, containing five different HES concentrations (2.4, 9.6, 16.2, 36.3 and 28.8 µg kg^−1^), were detected by the strip and HPLC. HPLC was performed in accordance with the following conditions: the column was Diamonsil C18 (250 × 4.6 mm, 5 µm), the mobile phases were a 20 mmol l^−1^ phosphoric acid and acetonitrile aqueous solution (60 : 40, V/V), the flow velocity was 0.5 ml min^−1^, the column temperature was held at 30°C, and the injection volume of the sample solution was 20 µl [36]. The test values by two methods were compared to the given concentrations by a one-sample *t*-test, and the test values of the two methods were also compared by an independent-sample *t*-test. The differences between the test values and the relative given concentrations were statistically significant at *p* < 0.05, the same as the differences between the relative test values of the two methods.

## 3. Results and discussion

### 3.1. Synthesis of hexoestrol immunogen

**Figure 1.**
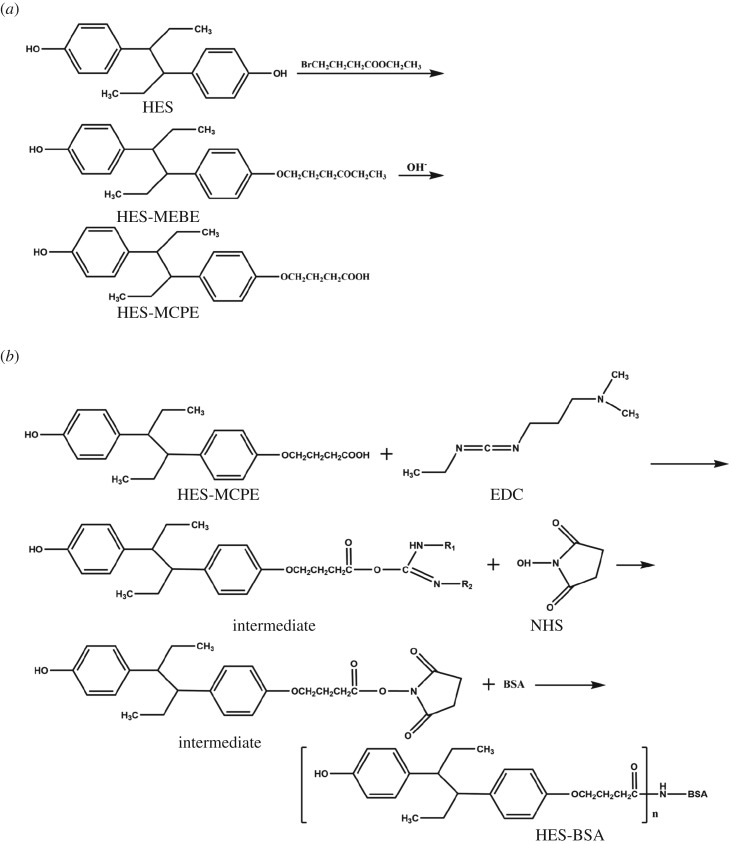
(*a*) Chemical structure of the synthesis process to HES-MCPE. (*b*) Via the active ester method, chemical strutures of the synthesis process to HES-BSA.

The immunogen HES-BSA for immunization of mice and the coating antigen HES-OVA in ELISA were synthesized from HES-MCPE, a derivative of HES according to oxidation reaction of 4-bromobutyric acid ethyl ester. The brief process for synthesis is outlined in figure 1*a*. The structure of HES-MCPE was identified by MS, and the free amino groups of BSA and the carboxyl group of HES-MCPE were conjugated by an active ester method, producing the immunogen (HES-BSA) and the coating antigen (HES-OVA) (figure 1*b*). The conjugation ratios of HES-MCPE to BSA and OVA were about 18.4 : 1 and 14.6 : 1, respectively. The BALB/c mouse, immunized by HES-BSA and showing highest titer (1 : 5.12 × 10^4^) and lowest IC_50_ values (19.93 µg l^−1^), was chosen for subsequent cell fusion and mAb preparation.

### 3.2. Production and properties of monoclonal antibody

After 9 days, growing hybridomas in almost every microtiter plate well could be observed. Via an indirect ELISA, OD_450 nm_ of the supernatants of each well was screened. The larger value cells (OD_450 nm_ > 2.0) were tested by a *ci-*ELISA to obtain the least IC_50_ of the supernatants. The re-cultured hybridomas 1C4, 3D6, 3E4, 4B9 were injected intraperitoneally into the BALB/c mouse to collect ascites fluid containing anti-HES mAb. The titre of HES-mAb was 1 : 1.28 × 10^5^, 1 : 5.12 × 10^5^, 1 : 2.56 × 10^5^ and 1 : 2.56 × 10^5^, respectively; the affinity constant (*K*_a_) was 9.0 × 10^9^, 4.5 × 10^10^, 3.2 × 10^10^ and 2.7 × 10^10^ l mol^−1^, respectively; the IC_50_ value was 1.903, 1.635, 1.768 and 1.792 ng ml^−1^; and the subclass of all anti-HES mAb was IgG1. The mAb 3D6, having both the highest titre and the lowest IC_50_, was chosen to be labelled by colloidal gold.

### 3.3. Optimization of the strip

To obtain the best performance of the strip, several conditions were optimized as follows: the pH value of the colloidal gold was 8.2, the concentration of anti-HES mAb labelled with colloidal gold was 5.7 µg ml^−1^, the colloidal gold-labelled HES mAb was sprayed onto the conjugate pad at thickness of 15 µl cm^−1^. In addition, HES-BSA and goat anti-mouse IgG were coated on NC membrane at a rate of 0.8 and 0.8 mg ml^−1^, respectively.

### 3.4. Sensitivity of the strip

**Figure 2.**
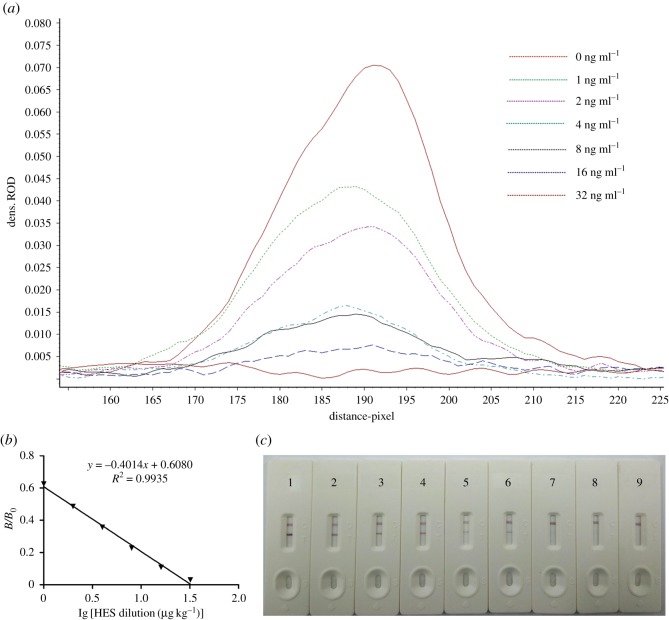
(*a*) ROD curves of fish samples. The extracts added with HES standard solutions at 0, 1, 2, 4, 8, 16 and 32 ng ml^−1^ were tested using the test strips. Test lines were scanned with a TSR3000 membrane strip reader. (*b*) Standard curve for HES using test strip detection. The *x*-axis represents the log concentration. *B*/*B*_0_ represents the percentage of ROD of standards divided by that of the ROD at 0 ng g^−1^. The linear regression correlation coefficient (*R*^2^) is 0.9935. (*c*) Standard HES were added in fish samples tested using the LFIST and the test lines were observed with the naked eye. LFIST 1–9 have the following HES concentrations (from left to right): 0, 1.0, 2.0, 4.0, 8.0, 16.0, 32.0, 40.0 and 48.0 µg kg^−1^.

Reference solutions of HES at concentrations of 0, 1.0, 2.0, 4.0, 8.0, 16.0, 32.0, 40.0 and 48 µg kg^−1^ were analysed using the LFIST. The results are shown in figure 2*a* and quantified in table 1. The ROD of the test line was measured using a TSR3000 Membrane Strip Reader (Bio-Dot), and plotted as a function of various concentrations of HES. A quantitative calibration curve was constructed by drawing the logarithmic values of scanned peak area values against the logarithmic concentrations of HES (figure 2*b*). According to the linear equation, the calculated IC_50_ was 1.86 ng ml^−1^; the linearity of the relationship was determined by the coefficient of determination (*R*^2^ = 0.9935). The LOD of the test strip was 0.62 µg kg^−1^ by the test strip reader and was 32 µg kg^−1^ by unaided visual assessment (figure 2*c*).

**Table 1.** *G*/peak and *G/D* × area of the ROD of test lines of HES in fish samples.

HES concentration (µg kg^−1^)	*G*/*D* × area-ROD (pixel)	*G*/peak-ROD (pixel)
0	98.5671	0.0708
1	61.5857	0.0433
2	47.9036	0.0339
4	35.1884	0.0166
8	23.4590	0.0143
16	10.7438	0.0072
32	3.0556	0.0035

In a previous study, anti-HES polyclonal antibody was prepared and applied in a one-step membrane-based competitive colloidal gold-based immunoassay in lateral flow format for the determination of HES in swine urine and liver [37]. However, the sensitivity of the test strip was far higher than 30 µg kg^−1^ by unaided visual assessment, which is much higher than ours, suggesting the strip developed in this study is more sensitive.

### 3.5. Specificity of the strip

To identify the specificity of the strip, fish samples were doped with HES and other competitors. Competitors including diethylstilbestrol, dienestrol, bisphenol A, estriol, oestradiol and provera were added at a concentration of 1000 µg kg^−1^. It was found that the strip gave a 11.63% CR with diethylstilbestrol, less than 0.9% with dienestrol, and less than 0.09% with other compounds (table 2). Therefore, the test strip for HES was highly specific and showed negligible CR with provera, estradiol, bisphenol A and estriol.

**Table 2.** CR of test strip with competitors.

compounds	IC_50_ (µg kg^−1^)	CR (%)
hexoestrol (HES)	1.86	100
diethylstilbestrol	16	11.63
dienestrol	>256	<0.9
bisphenol A	>2.0 × 10^3^	<0.09
oestradiol	>2.0 × 10^3^	<0.09
estriol	>2.0 × 10^3^	<0.09
provera	>2.0 × 10^3^	<0.09

### 3.6. Reproducibility of hexoestrol in fish samples

To determine the reproducibility of the strip test, fish samples containing 4.0, 12.0 and 24.0 µg kg^−1^ of HES were tested using three different batches of strips. As shown in table 3, for intra-assay and inter-assay reproducibility, recoveries were from 86.3 to 92.3% and from 85.8 to 93.4%, respectively. The intra- and inter-assay CV of the LFIST were both less than 6.0% (table 3). These data indicated that the results given by the strip test were highly reproducible.

**Table 3.** Recovery and intra-assay and inter-assay precision of the test strips for HES spiked in fish samples. (Fish samples were spiked with HES at 4, 12 and 24 µg kg^−1^ were tested using the strips. Intra-assay precision was estimated by using one batch of the test strips (*n* = 6). For inter-assay precision, three batches of the test strips were used to detect the given samples. The recoveries and CV were calculated from triplicate assays in all cases.)

spiked HES (µg kg^−1^)	intra-assay			inter-assay		
	mean ± s.d. (µg kg^−1^)	recovery (%)	CV (%)	mean ± s.d. (µg kg^−1^)	recovery (%)	CV (%)
4	3.45 ± 0.16	86.3 ± 4.0	4.64	3.43 ± 0.17	85.8 ± 4.3	4.96
12	10.65 ± 0.31	88.7 ± 2.6	2.91	11.02 ± 0.57	91.8 ± 4.8	5.17
24	22.15 ± 0.86	92.3 ± 3.6	3.88	22.41 ± 0.95	93.4 ± 4.0	4.24
						

### 3.7. Authenticity of the strip

A comparison between the strip test and HPLC was performed at five levels with fish samples. The results showed that although the test values by strip and by HPLC were all lower than the relative given concentration, there were no significant differences between test values and the given concentrations (*p* > 0.05). Moreover, there were no significant differences of the relative test values whether by strip or by HPLC (*p* > 0.05) (table 4).

**Table 4.** Comparison of the strip test with HPLC for three levels of HES residues in fish samples. (*n* = 6 per level of HES in fish samples.)

level of HES (µg kg^−1^) in fish samples	test strip (µg kg^−1^)	HPLC (µg kg^−1^)
2.4^a^	2.01 ± 0.11^a^	2.06 ± 0.07^a^
9.6^a^	8.11 ± 0.27^a^	8.26 ± 0.17^a^
16.2^a^	14.16 ± 0.46^a^	14.60 ± 0.28^a^
28.8^a^	25.44 ± 0.81^a^	26.21 ± 0.38^a^
36.3^a^	32.56 ± 0.99	33.12 ± 0.56

^a^For within-column comparisons, superscripts indicate no significant difference (*p* > 0.05).

### 3.8. Advantages and development trends of immunoassay test strips

In China, test strips are widely used in experiments in the field of food safety inspection. Instrumental analysis cannot deal with so many samples. The strip can really promote the progress of food safety inspection technology, achieving universal inspection. Unfortunately, large-scale instrument inspections cannot meet universal inspection requirements in terms of time, place and economy. Multi-target, high-throughput and digitization are the future development trend of the test strip. There are two technical approaches to achieving multi-target and high-throughput detection. Firstly, the antibodies against a common epitope of a certain class of targets are produced and a single test line is used to achieve the joint detection of multiple similar targets. Secondly, the chips of the test strips are designed. Multi-detection lines or a matrix are used to achieve the joint inspection of multiple similar or different targets. Digitization is the realization of quantitative detection. The development of portable card readers compensates for the arbitrariness of human eye observation and the non-portability of current card reading devices, and realizes digitized detection under certain conditions [30,38].

## 4. Conclusion

The use of a high-affinity mAb against HES enables us to establish a colloidal gold-based LFIST for detecting HES in fish meats. The major advantages of the one-step strip test are rapidity (less than 10 min), integration (all needed reagents were integrated in the strip), sensitivity, specific and cost-effectiveness. Therefore, the strip test holds great potential as an on-site screening tool for monitoring HES residues in fish meats.

## Data accessibility

Our data are deposited at Dryad: http://dx.doi.org/10.5061/dryad.92bh000 [39].
